# Integrated Profiling of DEHP-Induced Hippocampal Neurotoxicity in Adult Female Rats Based on Transcriptomic and Neurobiological Analyses

**DOI:** 10.3390/toxics14010079

**Published:** 2026-01-14

**Authors:** Jing Bai, Jiayu Li, Lei Tang, Wuxiang Sun, Fujia Gao, Xin Zhang, Rui Bian, Ruimin Wang

**Affiliations:** 1 Key Laboratory of Dementia and Cognitive Dysfunction, School of Public Health, North China University of Science and Technology, Tangshan 063210, China; baijing7858@163.com (J.B.); gaofujia1983@163.com (F.G.);; 2 School of Basic Medical Sciences, North China University of Science and Technology, Tangshan 063210, China; lijiayu010210@163.com (J.L.);; 3 School of Pharmacy, North China University of Science and Technology, Tangshan 063210, China; tl18332750192@163.com; 4 Department of Medicine, Yangzhou University, Yangzhou 225009, China

**Keywords:** Di-2-ethylhexyl phthalate, oxidative stress, mitochondrial dysfunction, adult neurogenesis, glial dysregulation

## Abstract

Di-2-ethylhexyl phthalate (DEHP) is a widely used plasticizer with recognized sex-dependent neurotoxicity. However, research on adult neurotoxicity is scarce, especially in females. In this study, adult female rats were exposed to a high-dose experimental model of DEHP (500 mg/kg/day) for 28 days to systematically evaluate hippocampal neurotoxicity. We found that DEHP exposure significantly impaired spatial learning and memory. Transcriptomics revealed enrichment in oxidative stress, complement activation, and neurodegenerative pathways. Specifically, cellular and molecular analyses showed that DEHP induced mitochondrial structural defects and elevated markers of oxidative damage (8-OHdG and 3-NT). While the upregulation of mitochondrial and antioxidant proteins (COX4I1, SOD2, and NQO1) indicated an attempted compensatory response, it remained inadequate to restore redox homeostasis. Under this neurotoxic microenvironment, DEHP triggered early neurogenesis, marked by the upregulation of SOX2 and DCX; however, NeuN levels remained unchanged, suggesting that this compensatory effort failed to expand the mature neuronal population. Ultimately, these pathological processes culminated in neurodegeneration, as evidenced by reduced synaptic proteins, suppressed Olig1/2 expression, and increased tau phosphorylation. Collectively, this study provides a comprehensive neurotoxic profile of DEHP in adult female rats, filling a research gap in this field.

## 1. Introduction

Di-(2-ethylhexyl) phthalate (DEHP) is currently one of the most widely used plasticizers worldwide. It is found extensively in medical devices, food packaging, building materials, children’s toys, and personal care products [1]. Because DEHP is bound to polymers mainly through physical interactions rather than chemical bonds, it easily migrates from materials during manufacturing, heating, or daily use, resulting in contamination across almost all environmental media [2]. Biomonitoring studies have detected DEHP and its metabolites in human blood, urine, and even brain tissue [3]. Population biomonitoring data indicated significant differences in DEHP exposure levels across age groups and between sexes. In many countries, internal levels of DEHP metabolites are usually higher in children than in adults, which is related to their frequent hand-to-mouth behaviors and greater contact with DEHP-containing products [4]. Meanwhile, multiple studies have found that adult women generally show higher DEHP exposure levels than men, possibly due to higher use of personal care products, differences in metabolic characteristics, and variations in hormonal regulatory mechanisms [5,6,7].

In humans, the major route of DEHP exposure is dietary intake, followed by dermal contact and inhalation [8]. Studies from China, Italy, Iran, Brazil, India, and Mexico have shown that DEHP exposure levels have exceeded the tolerable daily intake (TDI) of 50 μg/kg bw/day set by the European Food Safety Authority (EFSA) [9]. In addition, differences in DEHP use across countries and regions are substantial [10], leading to 2–3 orders of magnitude variation in urinary metabolite levels between high-exposure and low-exposure individuals [11]. Among occupational populations, one study reported that workers engaged in producing polyvinyl chloride (PVC) flooring exhibited urinary DEHP metabolite levels approximately 100 times higher than unexposed workers [12]. Although many countries have implemented regulations restricting DEHP use, limitations associated with cost and the performance of alternative plasticizers mean that no complete replacement for traditional DEHP is currently available.

As a prominent environmental endocrine disruptor, DEHP has gained increasing attention for its potent neurotoxicity. Existing research on DEHP-related neurotoxicity has focused primarily on transgenerational or developmental exposures and shows marked sex differences [13,14]. Previous studies have shown that prenatal DEHP exposure results in more severe spatial memory impairment in male ICR mice than in females [15]; early-life intraperitoneal injection of DEHP affects adult hippocampal development, with males being more significantly affected, whereas females exhibit a certain degree of protection [16]. In human populations, prenatal DEHP exposure has been linked to increased autism-related traits in young children, and early childhood exposure has been associated with increased autism-related traits at school age, particularly in boys [17]. These findings suggest that the neurotoxic effects of DEHP are strongly sex-dependent.

While the transgenerational and developmental neurotoxicity of DEHP is well-documented, its impact on the adult brain remains comparatively under-investigated. To date, evidence in this area has been derived almost exclusively from male models. Research has shown that adult male rats exposed to DEHP exhibited anxiety-like behaviors and cognitive impairment [18]. Similarly, in adult male C57BL/6J mice, DEHP induced oxidative stress in the brain, leading to increased locomotor activity and anxiety-like behavior [19]. Furthermore, DEHP was shown to compromise physiological integrity by increasing blood–brain barrier permeability and inducing inflammation-related changes in the male brain [20]. This male-centric body of evidence leaves a critical knowledge gap regarding the female neurotoxicological profile, hindering a comprehensive assessment of the health risks posed by DEHP exposure.

To bridge this critical knowledge gap, the present study employed a multi-dimensional approach integrating behavioral testing, mRNA transcriptomic profiling, histological assessment, and molecular biological analysis to systematically evaluate the neurotoxic effects of DEHP exposure in adult female rats. Specifically, we utilized a high-dose experimental model (500 mg/kg/day). The selection of this dose and exposure duration was based on a conventional high dose frequently utilized in established DEHP neurotoxicity research to facilitate the investigation of underlying pathological mechanisms [14]. Moreover, this dose was also adopted based on established research demonstrating clear neurobiological and behavioral impairments in adult male rats under comparable subchronic conditions [18], allowing for a direct cross-sex comparison. Ultimately, this study aims to comprehensively assess the neurotoxicity of DEHP and provide essential evidence regarding the sex-dependent effects of this widely used environmental chemical.

## 2. Materials and Methods

### 2.1. Animals and DEHP Exposure

Adult female Sprague-Dawley rats (8 weeks, 180 ± 20 g) were obtained from Beijing HFK Bioscience Co., Ltd. (Beijing, China; License No. SCXK 2019-0008). Animals were housed under standard conditions (23 ± 2 °C, 55 ± 10% humidity, 12 h light/dark cycle) with free access to food and water. After two weeks of acclimatization, rats were randomly assigned to a control group and a DEHP-exposed group (*n* = 10 per group) using a computer-generated random number table to ensure unbiased allocation. DEHP (Sigma-Aldrich, St. Louis, MO, USA; purity ≥ 99.5%) was dissolved in corn oil and administered by oral gavage at 500 mg/kg body weight once daily for 28 consecutive days. Animals in Ctrl group received an equal volume of corn oil. To ensure the objectivity of the results, all behavioral assessments and subsequent biochemical analyses were performed by researchers who were blinded to the treatment assignments.

### 2.2. Morris Water Maze Test

Hippocampal-dependent spatial learning and memory were assessed using the Morris water maze (MWM) on the second day after DEHP exposure. The apparatus consisted of a circular pool (150 cm in diameter, 60 cm in height) filled with water made opaque with nontoxic white paint and maintained at 22 ± 1 °C. A circular escape platform (10 cm in diameter) was submerged 1–2 cm below the water surface in a fixed quadrant. Extra-maze visual cues were placed on encircling curtains around the pool and kept constant throughout testing. Rats underwent one acquisition trial per day for three consecutive days. At the beginning of each trial, the animal was gently placed into the water facing the pool wall from one of the four starting positions. The trial ended when the rat found and climbed onto the platform or when 60 s had elapsed. Rats that failed to find the platform within 60 s were guided to it and allowed to remain on the platform for 20 s. Escape latency and swim speed were recorded with a video tracking system (ANY-maze, version 6.0). On day 4, a probe trial was carried out to evaluate memory retention. The platform was removed, and each rat was allowed to swim freely for 60 s. The time spent in the target quadrant, number of crossings over the previous platform location, and representative trajectories were recorded and analyzed. To minimize hormonal variability, estrous stages were assessed via vaginal smears immediately after testing. Only rats in diestrus or metestrus were included in the analysis.

### 2.3. Tissue Collection

Within 24 h following the completion of the behavioral tests, rats were deeply anesthetized with isoflurane and transcardially perfused with ice-cold PBS (pH 7.4) to clear the circulation, followed by sacrifice via decapitation. Animals (*n* = 10 per group) were allocated into four subsets: (1) mRNA sequencing (*n* = 3); (2) Western blot (left hippocampus) and IF (right hemisphere, 4% paraformaldehyde) (*n* = 3); (3) transmission electron microscopy (TEM; left CA1, 2.5% glutaraldehyde) and Nissl/FJB staining (right hemisphere, 4% paraformaldehyde) (*n* = 3); and (4) backup animal (*n* = 1) served as a biological reserve for methodological optimization. For the quantification of histological data, one section per animal was selected at a standardized anatomical level. To ensure comparability, images were captured from consistent coordinates within the hippocampal CA1, CA3, and DG regions.

### 2.4. Nissl Staining

Nissl staining was performed to evaluate the overall neuronal morphology and survival by labeling Nissl bodies in the cytoplasm [21]. Tissue sections (25 μm thick) were stained using the Cresyl Violet method. Briefly, sections were deparaffinized and rehydrated through graded ethanol solutions. Slides were then incubated in 0.1% Cresyl Violet Acetate solution for 10 min. Differentiation was performed in 95% ethanol containing 0.1% acetic acid under microscopic observation until the Nissl bodies were clearly defined and the background was adequately cleared. Finally, sections were dehydrated, cleared in xylene, and mounted with Neutral Balsam. Images were acquired using a brightfield light microscope (XS-213-011091, Nanjing Ruiyuan Optical Co., Ltd., Nanjing, China) and subsequently analyzed with Image J (Fiji distribution, NIH), version 1.52p.

### 2.5. Fluoro-Jade B Staining (FJB) Staining

FJB is a widely used, highly sensitive fluorescent method that specifically labels degenerating neurons. It strongly binds to positively charged molecules in degenerating neurons, distinguishing them from healthy cells. It is used to detect drug-induced neuronal damage [22]. Tissue sections (25 μm thick) were processed for FJB histochemistry. Slides were first immersed in a 0.06% KMnO_4_ solution for 10 min. After rinsing in distilled water, the sections were then incubated in the FJB working solution (0.0004% FJB in 0.1% acetic acid vehicle) for 20 min in the dark. Slides were then rinsed three times with distilled water. Finally, sections were cleared in xylene and mounted with DPX. FJB-positive neurons, characterized by bright green fluorescence in the cell bodies and processes, were visualized using a confocal microscope (Andor Dragonfly, Andor Technology, Belfast, UK) with an excitation wavelength of 480 nm and an emission wavelength of 525 nm, and subsequently analyzed with Image J.

### 2.6. TEM

The fixed tissue blocks were subjected to postfixation in 1% osmium tetroxide for 2 h. After thorough rinsing, tissues were dehydrated in a graded ethanol series and embedded in epoxy resin. Ultrathin sections (70 nm) were cut with an ultramicrotome, mounted on copper grids, stained with uranyl acetate and lead citrate, and examined under a transmission electron microscope (HT7650, Hitachi Hi-Tech, Tokyo, Japan). Mitochondrial morphology in the hippocampal CA1 region was evaluated. 

### 2.7. RNA Extraction and mRNA Sequencing

Total RNA was extracted from hippocampal tissue using TRIzol reagent (Ambion, Austin, TX, USA) according to the manufacturer’s instructions. RNA concentration and purity were determined using a Nanodrop 2000 spectrophotometer (Thermo Fisher Scientific, Waltham, MA, USA), and integrity was evaluated using an Agilent 5300 Fragment Analyzer (Agilent Technologies, Santa Clara, CA, USA). Only high-quality RNA sample (OD260/280 = 1.8~2.2, OD260/230 ≥ 2.0, RQN ≥ 7, 28S:18S ≥ 1.0, >1 g) was used to construct sequencing library. 

RNA sequencing and data analysis were performed by Hangzhou Cosmos Wisdom Biotech Co., Ltd. (Hangzhou, China). Briefly, 1 μg of total RNA was used to construct the transcriptome library using the Illumina Stranded mRNA Prep Ligation kit (Illumina, Inc., San Diego, CA, USA). Messenger RNA was enriched via polyA selection, fragmented, and reverse-transcribed into cDNA using random hexamer primers. Following end-repair and A-tailing, the libraries were size-selected for ~300 bp fragments and amplified via 15 PCR cycles. Sequencing was conducted on a NovaSeq X plus platform (2 × 150 bp paired-end). Raw data were processed by fastp to remove low-quality reads. The clean reads were aligned to the reference genome using HISAT2 and assembled by StringTie. Gene expression levels were quantified as transcripts per million (TPM) using RSEM. Differentially expressed genes (DEGs) were identified using DESeq2 (*p*.adjust ≤ 0.05), and functional enrichment analyses (GO and KEGG) were performed using Goatools (version 1.1.6) and KOBAS (version 3.0), respectively (Bonferroni-corrected *p* ≤ 0.05).

### 2.8. IF Staining

Brains were fixed cryoprotected in 30% sucrose in PBS until they sank. Coronal sections containing the hippocampus (25 µm) were cut on a cryostat and collected in PBS. After washing, sections were permeabilized with 0.1% Triton X-100 and blocked with 10% donkey serum for 1 h at room temperature. Sections were then incubated overnight at 4 °C with primary antibodies diluted in blocking buffer. Following PBS washes, sections were incubated with appropriate fluorophore-conjugated secondary antibodies (Alexa Fluor 488/594) for 2 h at room temperature in the dark. Nuclei were counterstained with DAPI. Images were acquired using a confocal microscope (Andor Dragonfly), and image analysis and three-dimensional reconstruction were performed using Imaris (version 9.5.0). Subsequently, quantification of fluorescence intensity was carried out using Image J. The antibodies used in IF Staining were as follows: Cytochrome c oxidase subunit 4 isoform 1 (COX4I1; 1:50; 66110; Proteintech Group, Inc., Rosemont, IL, USA), Superoxide dismutase 2 (SOD2; 1:50; sc-18503; Santa Cruz Biotechnology, Inc., Santa Cruz, CA, USA), 8-hydroxy-2′-deoxyguanosine (8-OHdG; 1:300; sc-65385; Santa Cruz Biotechnology), 3-nitrotyrosine (3-NT; 1:50; sc-32757; Santa Cruz Biotechnology), NAD(P)H quinone dehydrogenase 1 (NQO1; 1:50; sc-376023; Santa Cruz Biotechnology), Ionized calcium-binding adapter molecule 1 (Iba1; 1:50; NB100-1028; Novus Biologicals, Centennial, CO, USA), Complement component 3 (C3; 1:100; 21337-1-AP; Proteintech Group), Glial fibrillary acidic protein (GFAP; 1:1000; ab53554; Abcam, Cambridge, UK), SRY-box transcription factor 2 (SOX2; 1:500; ab97959; Abcam), Doublecortin (DCX; 1:50; sc-8066; Santa Cruz Biotechnology), Neuronal nuclei (NeuN; 1:100; 24307; Cell Signaling Technology, Danvers, MA, USA), Oligodendrocyte transcription factor 1 (Olig1; 1:50; GTX104823; GeneTex, Inc., Irvine, CA, USA), Oligodendrocyte transcription factor 2 (Olig2; 1:50; GTX132733; GeneTex), Proteolipid protein 1 (PLP1; 1:100; HA500202; Huabio, Hangzhou, China), p-tau231 (1:50; AP005; ABclonal Technology, Woburn, MA, USA), p-tau396 (1:100; AP1028; ABclonal Technology), Vesicle-associated membrane protein 2 (VAMP2; 1:250; ab181869; Abcam), Syntaxin (1:500; ab3265; Abcam), Synapse-associated protein 102 (SAP102; 1:30; PA1-045; Thermo Fisher Scientific, Inc., Waltham, MA, USA), Microtubule-associated protein 2 (MAP2; 1:50; sc-56561; Santa Cruz Biotechnology), Calcium/calmodulin-dependent protein kinase II α (CaMKIIα; 1:50; 50049; Cell Signaling Technology), Spinophilin (SPN; 1:500; 14136; Cell Signaling Technology), Alexa fluorTM488 donkey anti-rabbit IgG (1:250; A21206; Invitrogen; Thermo Fisher Scientific, Inc., Waltham, MA, USA), Alexa fluorTM 488 donkey anti-mouse IgG (1:250; A21202; Invitrogen), Alexa fluorTM 594 donkey anti-rabbit IgG (1:250; A21207; Invitrogen), Alexa fluorTM 594 donkey anti-mouse IgG (1:250; A21203; Invitrogen). 

### 2.9. Western Blot 

Hippocampal tissues were homogenized in ice-cold RIPA lysis buffer (HY-K1001; MedChemExpress, Monmouth Junction, NJ, USA) with a 10% protease inhibitor cocktail (HY-K0010; MedChemExpress). Lysates were incubated on ice for 30 min and centrifuged at 12,000× *g* for 15 min at 4 °C. The supernatants were collected, and protein concentration was determined using a BCA protein assay kit (P0012; Beyotime Institute of Biotechnology, Shanghai, China). Equal amounts of protein (20 µg per lane) were mixed with loading buffer, boiled for 5 min, separated by SDS–polyacrylamide gel electrophoresis (SDS–PAGE), and transferred onto PVDF membranes. After blocking with 5% BSA in TBST for 1 h at room temperature, membranes were incubated overnight at 4 °C with primary antibodies specific to the target proteins. After washing in TBST, membranes were incubated with HRP-conjugated secondary antibodies for 2 h at room temperature. Immunoreactive bands were visualized using a Super ECL Plus Kit (10010; Suzhou NCM Biotech Co., Ltd., Suzhou, China). and imaged with a digital imaging system (Bio-Rad Laboratories, Inc., Hercules, CA, USA). Band intensities were quantified using Image J and normalized to housekeeping proteins (α-tubulin or GAPDH). The antibodies used in Western blot were as follows: COX4I1 (1:2000); SOD2(1:200), 3-NT (1: 1500), NQO1(1:500), Iba1 (1:1000), C3 (1:1000), GFAP (1:1000), Olig1 (1:1000), VAMP2 (1:1000), Syntaxin (1:200), p-tau231 (1:800), p-tau396 (1:1000), SAP102 (1:300), Postsynaptic Density Protein 95 (PSD95; 1:1000; ab2723; Abcam), GAPDH (1:10,000; 60004; Proteintech Group), α-tubulin (1:10,000; ab7291; Abcam). 

### 2.10. Statistical Analysis

Data are expressed as mean ± standard error of the mean (SEM). Statistical analyses were performed using GraphPad Prism9.0. For comparisons between two groups, an unpaired Student’s *t* test was used; for multiple measurements over time in the Morris water maze, two-way repeated-measures ANOVA followed by post hoc tests (Bonferroni) was applied. A *p*-value < 0.05 was considered statistically significant.

## 3. Results

### 3.1. DEHP Exposure Induces Hippocampal Neuronal Injury and Spatial Learning and Memory Deficits in Adult Female Rats

**Figure 1:**
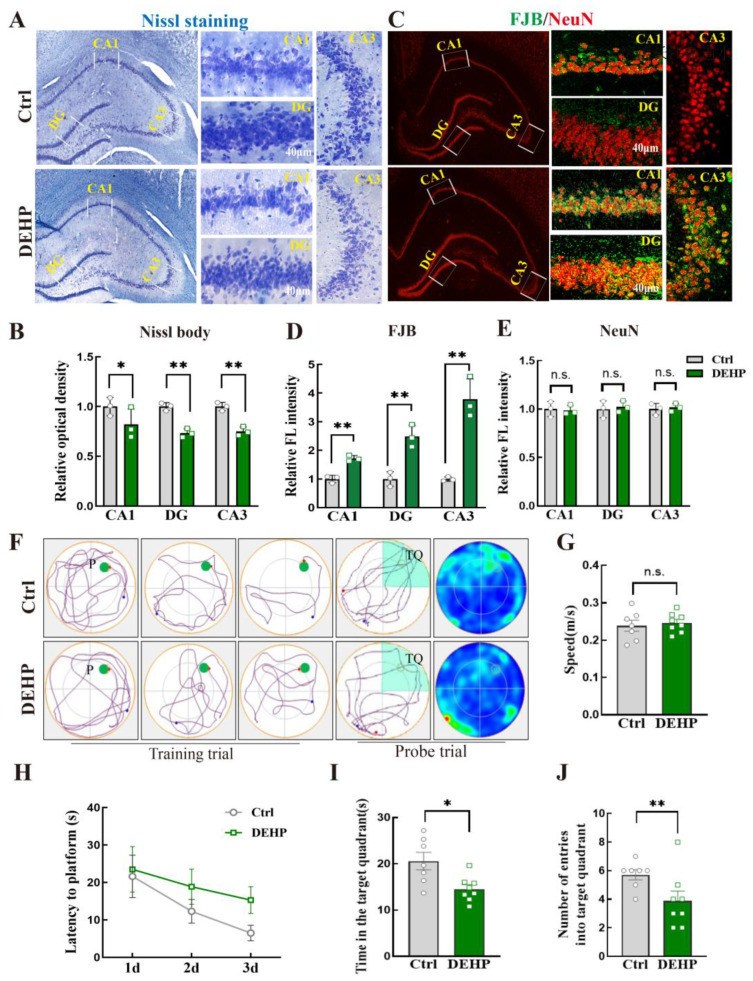
DEHP-induced hippocampal neuronal degeneration and spatial memory impairment in adult female rats. (**A**) Representative images of Nissl staining in the hippocampal CA1, DG, and CA3 regions of Ctrl and DEHP groups. (**B**) Relative optical density of Nissl bodies. (**C**) Representative double-immunofluorescence (IF) images of FJB and NeuN in the hippocampus. (**D,E**) Relative fluorescence intensity of FJB and NeuN. (**F**) Representative swimming trajectories during training trails and heatmaps during the probe trial in the Morris water maze. P, platform; TQ, target quadrant. Blue and red dots indicate the starting and ending positions, respectively. For heatmaps, the color gradient from blue to red represents increasing dwell time or occupancy frequency. (**G**) Average swimming speed. (**H**) Latency to find the hidden platform during the 3-day training phase. (**I**) Time spent in the target quadrant during the probe trial. (**J**) Number of entries into the target quadrant, *n* = 7–8. (Data are presented as mean ± SEM. * *p* < 0.05, ** *p* < 0.01; n.s. indicates no difference. *n* = 3 for Nissl, FJB, IF and Western blot; *n* = 7–8 for Morris water maze).

Nissl staining showed a reduced staining intensity in the CA1, DG, and CA3 regions of the DEHP-exposed rats compared with controls. The main features were a decrease in intracellular Nissl substance and a relatively sparse appearance, while the laminar structure remained intact (Figure 1A). Quantification confirmed lower optical density in all three regions (Figure 1B). FJB staining revealed more FJB-positive cells in the CA1, DG, and CA3 regions of the DEHP group, with significantly higher fluorescence intensity than controls (Figure 1C,D). In contrast, NeuN fluorescence intensity did not differ between the two groups in any of the three hippocampal regions (Figure 1C,E). 

In the Morris water maze, representative swimming tracks illustrated that the DEHP group exhibited poor directionality and dispersed search patterns compared to controls (Figure 1F). The swimming speed did not differ between groups (Figure 1G). During the training phase, the time to reach the platform was comparable on the first two training days, whereas DEHP-exposed rats showed a longer latency on day 3 (Figure 1H). In the probe trial, the DEHP group spent significantly less time in the target quadrant (Figure 1I) and entered the target quadrant fewer times than controls (Figure 1J).

### 3.2. DEHP Exposure Induces Widespread Transcriptomic Alterations Associated with Hippocampal Neurotoxicity in Adult Female Rats

**Figure 2:**
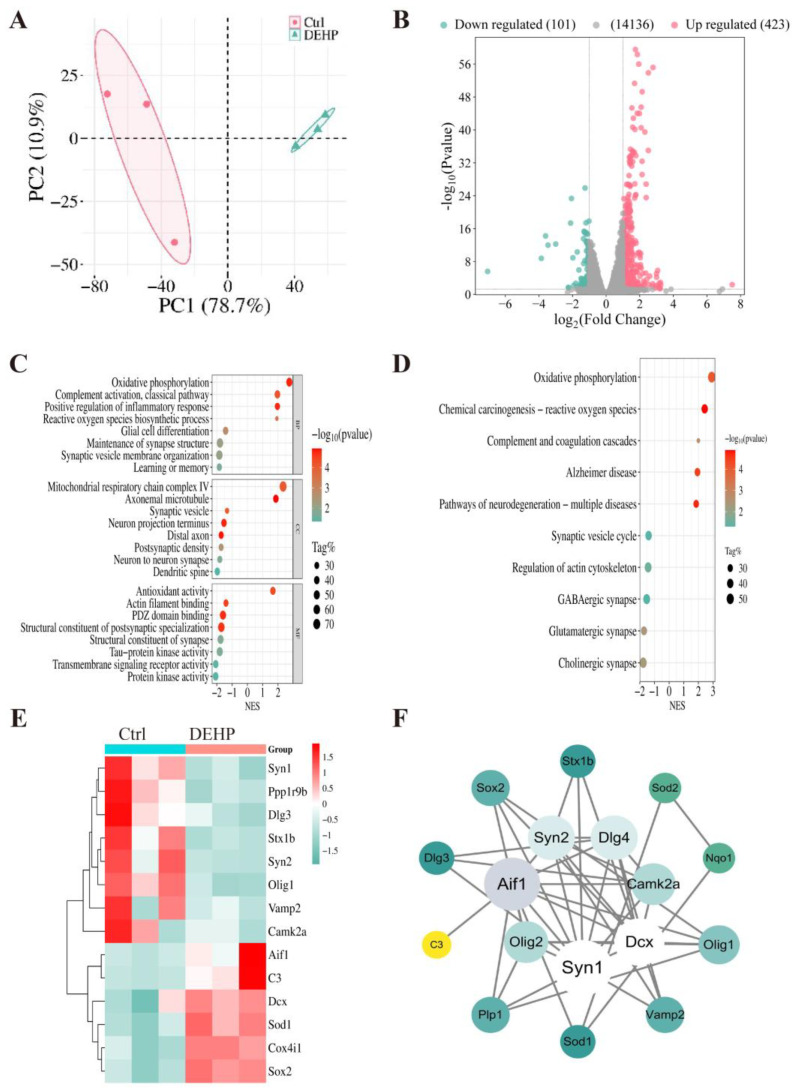
Transcriptomic analysis of the hippocampus following DEHP exposure. (**A**) PCA plot showing distinct clustering between Ctrl and DEHP groups. (**B**) Volcano plot of DEGs; red and green dots represent upregulated and downregulated genes, respectively. (**C**) GSEA analysis displaying representative GO terms associated with DEHP-induced neurotoxicity (categorized by BP, CC, and MF). (**D**) GSEA analysis highlighting key pathways related to neurotoxicity. (**E**) Heatmap of representative DEGs. (**F**) Protein–Protein Interaction (PPI) network of key identified targets. Node size and color represent the degree of connectivity (*n* = 3).

Principal Component Analysis (PCA) revealed a clear separation between the control and DEHP-exposed groups, indicating substantial differences in global transcriptional patterns (Figure 2A). To visualize the global gene expression alterations, a volcano plot was generated (Figure 2B). The analysis identified a total of 524 differentially expressed genes (DEGs) between the groups (Table S1). Specifically, 423 genes were significantly upregulated, while 101 genes were significantly downregulated. Gene Set Enrichment Analysis (GSEA) based on GO terms revealed a striking functional dichotomy across biological processes (Figure 2C, Table S2). Within the Biological Process (BP) category, terms such as “oxidative phosphorylation”, “complement activation”, “inflammatory response”, and “reactive oxygen species biosynthetic process” were significantly upregulated; conversely, terms including “glial cell differentiation”, “maintenance of synapse structure”, “learning or memory”, and “regulation of synaptic plasticity” were significantly downregulated. These findings were further corroborated by the Cellular Component (CC) and Molecular Function (MF) analyses: the CC analysis highlighted an enrichment of mitochondrial respiratory chain complexes alongside a reduction in postsynaptic density, while the MF analysis emphasized enhanced antioxidant activity and the suppression of structural constituents of postsynaptic specialization.

KEGG pathway analysis (Figure 2D, Table S3) further demonstrated that multiple signaling pathways associated with synaptic function, mitochondrial metabolism, and neural processes were altered. KEGG pathways related to mitochondrial energy metabolism and oxidative stress, specifically “Oxidative phosphorylation” and “Reactive oxygen species”, were significantly enriched with positive normalized enrichment scores. Furthermore, disease-associated pathways such as “Alzheimer disease” and “Pathways of neurodegeneration” were also upregulated. Conversely, pathways critical for neurotransmission, including “Synaptic vesicle cycle”, “GABAergic synapse”, “Glutamatergic synapse”, and “Cholinergic synapse”, showed significant negative enrichment. To characterize the molecular alterations associated with DEHP exposure, we analyzed the expression profiles of specific marker genes. The heatmap analysis (Figure 2E) demonstrated that genes related to synaptic function (*Syn1*, *Syn2*, *Stx1b*, *Dlg3*, *Vamp2*, *Ppp1r9b*, *Camk2α*) and oligodendrocyte differentiation (*Olig1*) were significantly downregulated in the DEHP-treated group compared to controls. In contrast, the expression levels of genes associated with oxidative stress (*Sod1*, *Cox4i1*), glial response (*Aif1*, *C3*), and neurogenesis (*Sox2*, *Dcx*) were significantly upregulated. Additionally, based on silico analysis, the protein–protein interaction (PPI) network (Figure 2F) visualized the potential connectivity among these proteins, highlighting Syn1, Aif1, and Dcx as the central hub nodes with the highest degree of interaction within the network.

### 3.3. DEHP Exposure Induces Mitochondrial Dysfunction and Oxidative Stress in the Hippocampus of Adult Female Rats

**Figure 3:**
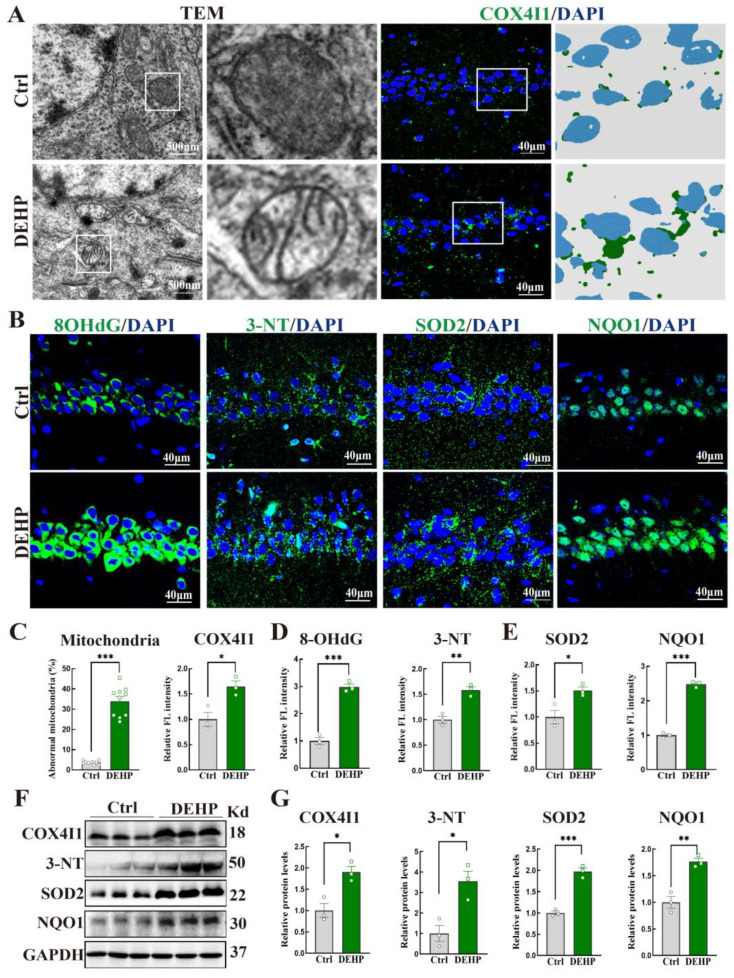
DEHP-induced mitochondrial dysfunction and oxidative stress in the hippocampus of adult female rats. (**A**) Representative Transmission Electron Microscopy (TEM) images showing mitochondrial ultrastructure and immunofluorescence (IF) images of COX4I1 with 3D surface rendering. The white boxes indicate magnified regions of interest (ROI). (**B**) Representative IF images of oxidative stress markers (8-OHdG, 3-NT) and antioxidant enzymes (SOD2, NQO1). (**C**) Quantification of the percentage of abnormal mitochondria and the relative fluorescence intensity of COX4I1. (**D**,**E**) Quantitative analysis of the relative fluorescence intensity for 8-OHdG, 3-NT, SOD2 and NQO1. (**F**) Representative Western blot bands for COX4I1, 3-NT, SOD2, and NQO1 proteins. GAPDH was used as a loading control. (**G**) Quantification of relative protein levels normalized to GAPDH. (Data are presented as mean ± SEM. * *p* < 0.05, ** *p* < 0.01, *** *p* < 0.001 vs. Ctrl; *n* = 3).

To evaluate the impact of DEHP on mitochondrial integrity and cellular redox status, we examined ultrastructural changes and oxidative stress markers. Ultrastructural analysis using transmission electron microscopy (TEM) revealed that mitochondria in the control group maintained a normal morphology with intact double membranes and dense cristae. In contrast, the DEHP-exposed group exhibited significant mitochondrial damage, characterized by swelling, vacuolization, and cristae disruption (Figure 3A). Quantitative analysis confirmed a significant increase in the percentage of abnormal mitochondria in the DEHP group (Figure 3C). Despite the structural impairment, the expression of the mitochondrial respiratory chain subunit COX4I1 was significantly upregulated, as evidenced by both IF (Figure 3A,C) and Western blot analysis (Figure 3F,G).

Consistent with mitochondrial dysfunction, DEHP exposure triggered severe oxidative stress. IF staining showed a marked increase in the levels of 8-OHdG and 3-NT in the DEHP group compared to controls (Figure 3B,D). The accumulation of 3-NT was further corroborated by Western blot analysis (Figure 3F,G). In response to this elevated oxidative stress, the antioxidant defense system was activated, as indicated by the significant upregulation of SOD2 and NQO1 protein levels in the DEHP-treated rats, confirmed by both IF (Figure 3B,E) and Western blot assays (Figure 3F,G).

### 3.4. DEHP Exposure Induces Microglial Activation and Astrocytic Dysfunction in the Hippocampus of Adult Female Rats

**Figure 4:**
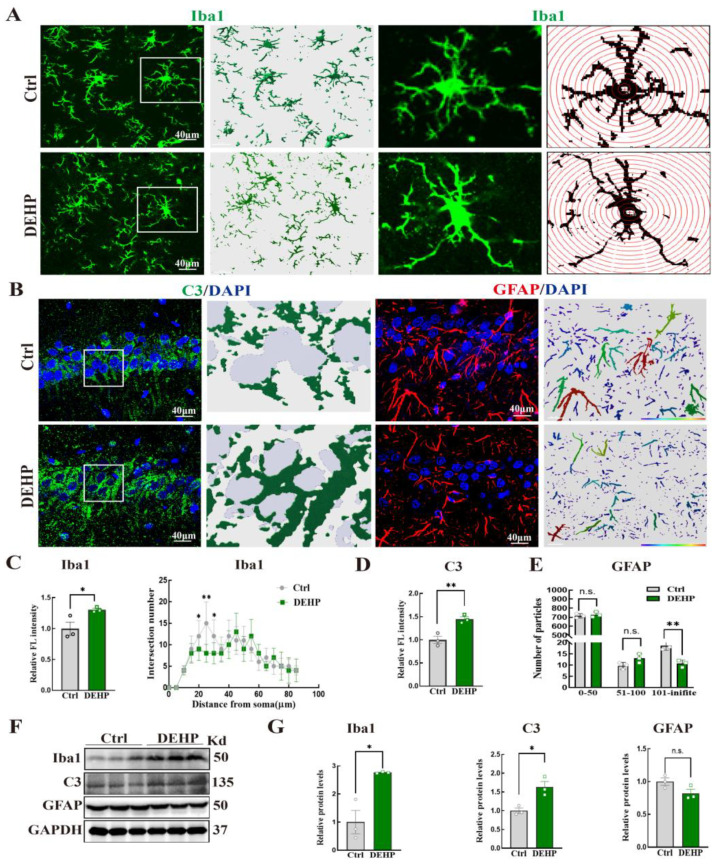
DEHP-induced microglial activation and astrocytic structural disintegration in the hippocampus of adult female rats. (**A**) Representative IF images of Iba1-positive microglia. Sholl analysis used to measure process ramification. The white boxes indicate magnified regions of interest (ROI). (**B**) Representative immunofluorescence (IF) images of C3 and GFAP in the hippocampus. 3D surface rendering was performed for C3, and different colors distinguish GFAP fragment sizes to assess structural integrity. The white boxes indicate magnified ROI. (**C**) Relative fluorescence intensity of Iba1 and Sholl analysis quantification showing the number of intersections at varying distances from the soma. (**D**) Quantitative analysis of the relative fluorescence intensity of C3. (**E**) Quantification of GFAP-positive particle sizes. (**F**) Representative Western blot bands for Iba1, C3 and GFAP. GAPDH was used as a loading control. (**G**) Quantification of relative protein levels normalized to GAPDH. (Data are presented as mean ± SEM. * *p* < 0.05, ** *p* < 0.01; n.s., not significant. *n* = 3).

We first examined microglia, the resident immune sentinels. As shown in Figure 4A, microglia in the DEHP-exposed group exhibited a distinct morphological transition from a highly ramified, resting state to an ameboid-like activated phenotype, characterized by enlarged somata and retracted processes. Sholl analysis quantitatively confirmed this loss of complexity, revealing a significant reduction in the number of process intersections at defined distances from the soma (Figure 4C). Consistent with these morphological changes, the expression of the microglial marker Iba1 was significantly upregulated, as evidenced by both increased relative fluorescence intensity (Figure 4C) and elevated protein levels in Western blot analysis (Figure 4F,G).

We next examined the expression of C3 and the status of astrocytes. We observed a robust upregulation of C3 level in both IF (Figure 4B,D) and Western blot (Figure 4F,G). However, total GFAP protein levels remained statistically unchanged (Figure 4G). Notably, morphological assessment revealed severe fragmentation of astrocytic processes (Figure 4B). Quantitative particle analysis further corroborated this finding: while the number of small particles (0–50 pixels) was unchanged, there was a significant depletion of large particles (101–infinite pixels) in the DEHP group compared to controls (Figure 4E).

### 3.5. DEHP Dysregulates Hippocampal Neurogenesis Dynamics in Adult Female Rats

**Figure 5:**
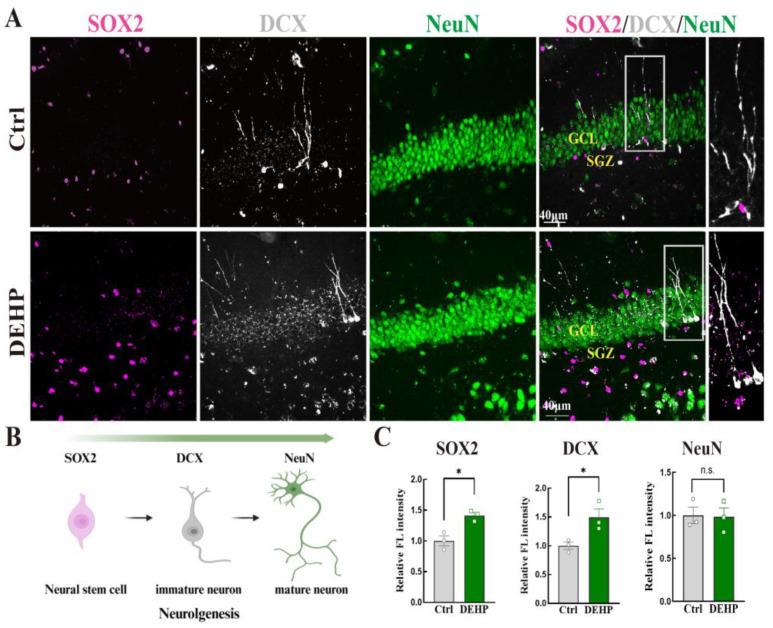
DEHP-induced aberrant hippocampal neurogenesis dynamics in adult female rats. (**A**) Representative immunofluorescence (IF) images of the neural stem cell marker SOX2 (magenta), the immature neuron marker DCX (white), and the mature neuron marker NeuN (green) in the hippocampal dentate gyrus. (**B**) Schematic of adult neurogenesis stages and stage-specific markers, with arrows indicating the progression from stem cells to mature neurons. (**C**) Quantitative analysis of the relative fluorescence intensity for SOX2, DCX, and NeuN. (Data are presented as mean ± SEM. * *p* < 0.05; n.s., not significant. *n* = 3).

Given the critical role of glial cells in the neurogenic niche, we evaluated the impact of DEHP on neurogenesis by examining markers corresponding to specific developmental stages (Figure 5B). As shown in Figure 5A, the fluorescence intensity of the neural stem cell marker SOX2 in the subgranular zone (SGZ) was visibly higher in the DEHP-exposed group, a finding confirmed by quantitative analysis (Figure 5C). Similarly, we observed a significant upregulation of the immature neuron marker DCX, with stronger signals extending into the granule cell layer (GCL) in the DEHP group (Figure 5A,C). In contrast to the early-stage markers, the expression of the mature neuronal marker NeuN remained unaltered, with no significant difference observed in fluorescence intensity between groups (Figure 5A,C).

### 3.6. DEHP Impacts Oligodendrocyte Differentiation, Tau Phosphorylation, and Synaptic Integrity

**Figure 6:**
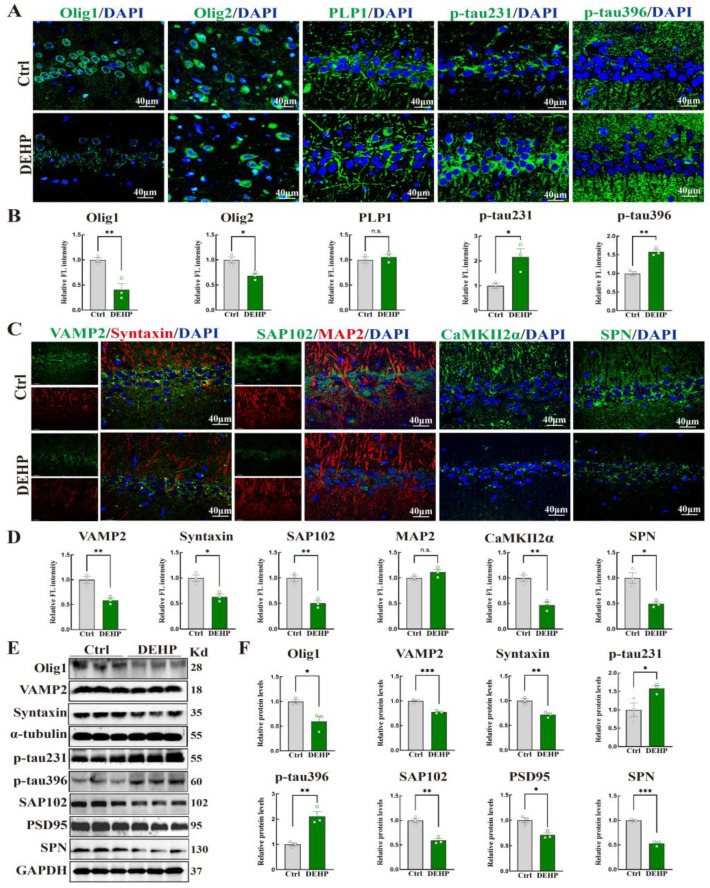
DEHP-induced impairment of oligodendrocyte homeostasis, tau hyperphosphorylation, and loss of synaptic integrity in the hippocampus of adult female rats. (**A,B**) Representative immunofluorescence (IF) images and quantification of oligodendrocyte lineage markers (Olig1, Olig2, PLP1) and phosphorylated tau (p-tau231, p-tau396). (**C,D**) Representative IF staining and quantitative analysis of pre-synaptic (VAMP2, Syntaxin) and post-synaptic/dendritic proteins (SAP102, MAP2, CaMKIIα, SPN). (**E**) Representative Western blot bands for Olig1, synaptic proteins, and tau phosphorylation markers. GAPDH or α-tubulin was used as a loading control. (**F**) Quantification of relative protein levels normalized to GAPDH or α-tubulin. (Data are presented as mean ± SEM. * *p* < 0.05, ** *p* < 0.01, *** *p* < 0.001 vs. Ctrl; n.s., not significant. *n* = 3).

To further assess the neuropathological alterations induced by DEHP, we examined the profiles of oligodendrocyte status, tau phosphorylation, and synaptic integrity. IF staining and Western blot analysis were performed to evaluate oligodendrocyte-related proteins. As shown in Figure 6A,B, the relative fluorescence intensities of Olig1 and Olig2 were significantly lower in the DEHP group compared to controls. Western blot data confirmed the downregulation of Olig1 protein levels (Figure 6E,F). Conversely, the fluorescence intensity of PLP1 was significantly higher in the DEHP group (Figure 6A,B). Simultaneously, we assessed tau phosphorylation. The fluorescence intensities (Figure 6A,B) and protein levels (Figure 6E,F) of both p-tau231 and p-tau396 were significantly increased in the DEHP group compared to the control group.

Finally, we quantified the expression of pre- and post-synaptic proteins as well as a dendritic marker. In the DEHP group, the expression levels of the pre-synaptic proteins VAMP2 and Syntaxin were significantly decreased in both IF (Figure 6C,D) and Western blot assays (Figure 6E,F). Similarly, regarding post-synaptic markers, IF analysis revealed a significant reduction in the fluorescence intensities of SAP102, CaMKIIα, and SPN (Figure 6C,D). Consistently, Western blot results confirmed the downregulation of protein levels for SAP102 and PSD95, as well as SPN (Figure 6E,F). However, the relative fluorescence intensity of the dendritic marker MAP2 showed no statistically significant difference between the DEHP and control groups (Figure 6C,D).

## 4. Discussion

In the present study, we provide integrated evidence characterizing the neurotoxic effects of subchronic DEHP exposure in adult female rats. While DEHP neurotoxicity has been extensively studied in the context of transgenerational and developmental exposure, or behavioral deficits in adult males [18,23,24], the potential impact on the adult female brain has remained largely underexplored. Our data demonstrate that the adult female hippocampus is a definite target for DEHP neurotoxicity. We show that DEHP exposure leads to spatial memory deficits and neuronal injury, driven by a multifaceted mechanism involving mitochondrial dysfunction, oxidative stress, glial responses, and synaptic failure. This study systematically maps the transcriptomic and neurobiological profiles of DEHP toxicity in females, contributing essential data to the understanding of chemical health risks.

The behavioral results confirm that subchronic exposure to DEHP (500 mg/kg) significantly impairs hippocampus-dependent spatial learning and memory in adult females. This functional deficit corresponds with the histological evidence of neuronal degeneration in the CA1, CA3, and DG regions. These findings establish a clear neurotoxic phenotype in the adult female hippocampus under the current exposure regimen. Notably, previous research on adult male rats has primarily attributed DEHP-induced neurotoxicity to generalized oxidative stress, blood–brain barrier dysfunction, and neuroinflammatory responses [25,26,27]. In contrast, our study in females highlights a distinct profile. While oxidative stress is a shared feature between sexes, our transcriptomic analysis revealed a prominent molecular signature characterized by the enrichment of “complement activation” and “glial cell differentiation” pathways. Since these specific neuroimmune profiles have rarely been emphasized in male DEHP studies. This underscores the necessity of future studies to directly compare sex-specific toxicological mechanisms.

The disruption of mitochondrial-redox homeostasis appears to be a central driver of the observed hippocampal injury. Ultrastructural analysis revealed severe mitochondrial swelling and cristae fragmentation within the hippocampal CA1 region. Such structural disintegration underpins the mitochondrial inefficiency that fuels the severe oxidative stress observed in this study. This is further evidenced by the significant accumulation of 3-NT and 8-OHdG, which serve as specific markers of protein nitration and DNA oxidation, respectively [28]. The presence of these biomarkers reflects a state of severe DEHP-induced oxidative and nitrative damage within the hippocampal tissue. Correspondingly, transcriptomic analysis highlighted ‘oxidative phosphorylation’ as a key enriched pathway, which was corroborated by the paradoxical upregulation of the electron transport chain subunit COX4I1 and antioxidant enzymes (SOD2, NQO1). COX4I1 serves as the key regulatory subunit of Cytochrome c Oxidase (Complex IV), the terminal and rate-limiting enzyme of the mitochondrial electron transport chain essential for ATP synthesis [29]. SOD2 functions as the primary mitochondrial scavenger, converting toxic superoxide radicals into hydrogen peroxide [30], while NQO1 acts as a versatile cytoprotective enzyme that maintains cellular redox homeostasis by preventing quinone-induced oxidative stress [31]. We propose that the simultaneous upregulation of COX4I1, SOD2, and NQO1 reflects a compensatory effort to maintain mitochondrial function and redox balance in response to the evident structural damage [32]. However, this response was likely overwhelmed by the magnitude of the DEHP-induced oxidative challenge. Ultimately, the severe structural and oxidative damage to mitochondria following DEHP exposure may act as a primary cause of neuronal degeneration in the hippocampal subregions.

Expanding beyond direct mitochondrial and oxidative damage, this neurotoxic microenvironment appears to drive a complex pattern of glial dysregulation. Regarding microglia, DEHP induced a classic pro-inflammatory state, evidenced by ameboid morphology and increased Iba1 expression. Given the established crosstalk between activated microglia and reactive astrogliosis [33], we further investigated the response of hippocampal astrocytes. The observed divergence between elevated C3 levels and the structural disintegration of astrocytes highlights a non-canonical glial response. Unlike classical hypertrophic astrogliosis, where GFAP expression typically increases alongside process extension, our findings revealed a degenerative state characterized by fragmented processes despite stable total GFAP levels. This specific morphological breakdown is histologically defined as clasmatodendrosis [34], a pathological change typically associated with severe mitochondrial failure and energy depletion [35]. Such structural deterioration, when coupled with the robust upregulation of C3, a hallmark of the neurotoxic “A1-like” reactive profile [36], suggests that DEHP may drive a unique form of glial dysfunction. Collectively, DEHP exposure triggers a coordinated glial response characterized by microglial activation and astrocytic impairment, which likely serves as a primary driver of hippocampal neuronal injury.

The hostile microenvironment, characterized by oxidative damage and glial dysregulation, potentially stimulates compensatory early neurogenesis. SOX2 is a high-mobility group transcription factor essential for maintaining the self-renewal and pluripotency of neural stem cells (NSCs), representing the initial proliferative pool [37]. DCX serves as a microtubule-associated protein exclusively expressed in migrating neuroblasts and immature neurons, marking the intermediate stage of expansion and neurite extension [38]. Finally, NeuN acts as a specific nuclear antigen for post-mitotic neurons, indicating successful functional maturation [39]. Our results revealed that DEHP exposure significantly upregulated early neurogenic markers (SOX2, DCX) while NeuN expression remained unaltered. This dissociation likely reflects an impaired transition from the differentiation phase to the maturation stage. Specifically, while the upregulation of SOX2 and DCX suggests that the hippocampus may have initiated compensatory proliferation in response to injury, the deterioration of the glial microenvironment potentially prevents these newborn cells from successfully surviving and maturing into NeuN-positive neurons. Research indicates that successful neurogenesis depends on a supportive neurogenic niche primarily maintained by healthy astrocytes and microglia [40]. The observed astrocytic clasmatodendrosis and microglial inflammation likely disrupt this niche, hindering the integration of newly generated immature neurons into functional neural circuits [41]. Furthermore, although NeuN levels remained unchanged, FJB staining confirmed early neurodegeneration. This may indicate that the concurrent upregulation of SOX2 and DCX represents a functional buffer that helps sustain the mature neuronal population and prevents massive loss during this subchronic exposure.

Ultimately, the various cellular and molecular disruptions discussed above converge to compromise synaptic integrity, which constitutes the structural basis of memory. We found widespread downregulation of both pre-synaptic (VAMP2, Syntaxin) and post-synaptic (PSD95, SAP102, CaMKIIα) proteins. VAMP2 and Syntaxin are core components of the SNARE complex, which is indispensable for synaptic vesicle fusion and neurotransmitter release [42]. On the post-synaptic side, PSD95 and SAP102 serve as critical scaffolding proteins that anchor glutamate receptors to the postsynaptic density, ensuring signal reception integrity [43], while CaMKIIα acts as a pivotal kinase driving long-term potentiation (LTP) and memory consolidation [44]. Consequently, the concurrent loss of these structural and functional pillars signifies a collapse in synaptic connectivity. This pattern might involve the complement system, although this remains a speculation based on our current data. In the complement pathway, C3 is thought to tag synapses for removal by microglial CR3 receptor [45]. Our findings of increased C3 levels, microglial activation, and decreased synaptic proteins are consistent with this possibility, though further evidence is required for confirmation. Furthermore, the increase in phosphorylated tau (p-tau231/396) suggests possible cytoskeletal instability. Hyperphosphorylated tau detaches from microtubules, potentially leading to transport failure of essential cargo (such as mitochondria and synaptic vesicles) to the synapse, which further exacerbates synaptic loss and neuronal dysfunction [46,47]. Additionally, Olig1 and Olig2 are key transcription factors essential for the differentiation of oligodendrocytes and the integrity of myelin sheaths [48]. In our study, the significant downregulation of Olig1 and Olig2, despite preserved PLP1 levels, suggests that the regenerative capacity of myelin are compromised. This impairment likely renders the hippocampus more vulnerable to further damage, contributing to cognitive decline [49].

Despite the insights gained from this study, several limitations should be acknowledged. First, while the current dosing regimen was instrumental for identifying key pathology and mechanistic hubs, it represents a relatively high-level subchronic exposure; thus, future studies incorporating a broader range of environmentally relevant concentrations are necessary to refine the toxicological risk assessment. Second, our findings are primarily based on morphological and molecular characterizations, and direct functional validation, such as electrophysiological recordings or cell-specific interventions, is required to establish a definitive causal link between the observed structural deficits and cognitive impairment.

## 5. Conclusions

**Figure 7:**
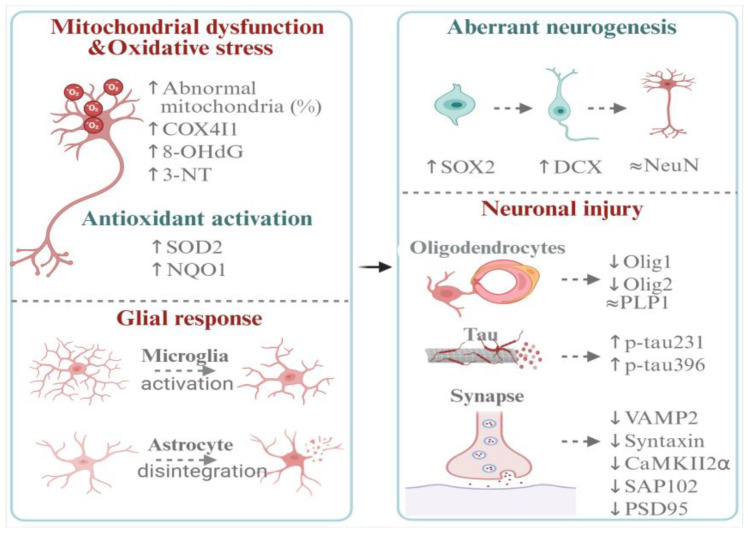
Schematic illustration of the proposed mechanism underlying DEHP-induced hippocampal neurotoxicity.

The findings of this study suggest that subchronic DEHP exposure, in this high-dose experimental model, impairs spatial learning and memory and induces hippocampal neuronal injury in adult female rats. Our integrated analyses indicate that these neurotoxic effects may be driven by mitochondrial dysfunction and oxidative stress, which are concurrent with microglial activation and astrocytic clasmatodendrosis. Furthermore, DEHP exposure appears to alter neurogenesis dynamics and impact oligodendrocyte differentiation, tau phosphorylation, and synaptic integrity. This study provides a comprehensive profiling of the potential hippocampal neurotoxicity induced by DEHP exposure in adult female rats (Figure 7).

## Data Availability

The raw data supporting the conclusions of this article will be made available by the authors on request.

## Supplementary Materials

The following supporting information can be downloaded at: https://www.mdpi.com/article/10.3390/toxics14010079/s1. Table S1. List of differentially expressed genes (DEGs) identified from the hippocampal transcriptomic analysis. Table S2. GO enrichment analysis results for the transcriptomic dataset. Table S3. Significant signaling pathways in the transcriptomic profile identified by KEGG analysis.
